# Type 1 diabetes does not impair the physical capacity of non-sedentary adolescents

**DOI:** 10.1186/s13098-017-0300-7

**Published:** 2017-12-16

**Authors:** Milena S. Nascimento, Carolina F. Espindola, Cristiane do Prado, Melina Blanco Amarins, Ana Lucia Potenza, Luciana Pacheco, Erica Santos, Teresa Cristina A. Vieira

**Affiliations:** 1 Departamento of Pediatrics, CTIP-Centro de Terapia Intensiva Pediátrica, Hospital Israelita Albert Einstein, Av. Albert Einstein, 627-701, São Paulo, 05651-901 Brazil; 2 Department of Pediatrics, Hospital Darcy Vargas, São Paulo, Brazil

**Keywords:** Diabetes mellitus type 1, Physical activity, Glycemic control

## Abstract

**Background:**

Type 1 diabetes patients have a higher risk of developing hypoglycemia or hyperglycemia during physical activity, which may compromise their safety during exercise but results regarding the exercise capacity of patients with type 1 DM when compared to control subjects have been contradictory.

**Aim:**

To evaluate if type 1 diabetes affects the capacity of adolescents to exercise.

**Methods:**

The study enrolled 37 adolescents in stage 2–4 of the Tanner scale, aged from 10 to 14 years, 21 with type 1 diabetes and 16 without any chronic diseases. All subjects performed an incremental submaximal exercise test in a cycle ergometer. At the end of every test stage, glycemia and blood lactate levels were measured. During the test, heart rate was monitored and the Borg rating of perceived exertion (RPE) was used to assess fatigue.

**Results:**

The two groups displayed no significant differences in anthropometric variables. The response to exercise, as evaluated by Borg RPE (p = 0.829), maximum oxygen uptake (VO_2_max) (p = 0.977), heart rate (p = 0.998), maximum load (p = 0.977), absolute load at lactate threshold (p = 0.377) and relative load at lactate threshold (p = 0.282), was also similar between the control and the type 1 diabetes group. Finally, there were no significant correlations between HbA_1c_ levels, VO_2_max, duration of disease and pre-test glycemia levels.

**Conclusions:**

We detected no significant differences in lactate threshold, VO_2_max and heart rate during exercise between healthy adolescents and non-sedentary adolescents with type I diabetes, indicating that both groups had similar physical fitness and, therefore, that type 1 diabetes is not an obstacle for physical activity. This study was approved by the ethical committee of the Hospital Israelita Albert Einstein (Ethical Committee Number: 53638416.9.0000.0071) and free and informed consent was obtained from all participants and their legal representatives.

## Background

Physical activity improves fasting blood glucose levels and insulin sensitivity in patients with type 1 diabetes (type 1 DM), leading to a reduction in the required daily dose of insulin. Furthermore, exercise also benefits the metabolic, cardiovascular and neuroendocrine systems, promoting weight loss, a reduction in fat accumulation and improving cardiovascular function and blood pressure. On the other hand, patients with type 1 DM have a higher risk of developing hypoglycemia or hyperglycemia during physical activity, which may compromise their safety during exercise [1, 2]. The balance between energy supply and consumption and the maintenance of euglycemia during exercise depend on a complex hormonal response that requires insulin, glucagon, cortisol, growth hormone, adrenaline and noradrenaline [3, 4]. Glycemia is also influenced by the predominant type of metabolism used in exercise, which can be anaerobic or aerobic, depending on the intensity of the physical activity as well as on the cardiovascular performance of the subject [5]. Therefore, it is important to evaluate the physical fitness of the patients before prescribing exercise [6, 7].

The incremental submaximal exercise test has been used to assess cardiovascular performance [8]. During this test, physical fitness is evaluated by determining parameters of physiological thresholds, such as maximum oxygen uptake (VO_2_max), or by measuring blood lactate and estimated VO_2_max [9]. VO_2_max corresponds to the moment when the maximum capacity to deliver oxygen for glucose oxidation is achieved. This defines the maximum limit for the aerobic exercise, also designated individual anaerobic threshold. Once this threshold is exceeded, there is an exponential increase in blood lactate (lactate threshold—LT) and a tendency for hyperglycemia. The persistence of physical effort after reaching the LT leads to loss of the oxidative capacity of the muscle, shifting the metabolism to anaerobic, which leads to lactate accumulation, faster fatigue, release of catecholamine and increase blood glucose levels [10, 11]. Physical fitness evaluation through blood lactate measurement, especially using portable devices, can easily be performed in field tests, besides being accessible to most physical evaluation professionals due to its relatively low cost [9]. In healthy subjects, measurement of blood lactate during exercise has been used to evaluate aerobic capacity and to determine the ideal intensity of exercise [12].

Since physical activity is essential for glycemic control in diabetic patients, it is extremely important that their exercise capacity is evaluated. The American College of Sports Medicine recommends that diabetic patients exercise as healthy subjects, do but results regarding the exercise capacity of patients with DM1 when compared to control subjects have been contradictory [13]. Performing a submaximal exercise test in patients with DM1 allows the evaluation of the metabolic response of these patients in a controlled environment, helping define the ideal intensity of physical activity for these patients. This can minimize the risks of hypoglycemia or hyperglycemia and favour patient engagement in physical activity practice.

The hypothesis of the present study is that well controlled DM1 subjects and controls submitted to the incremental submaximal exercise test will reach the LT at higher load levels than uncontrolled DM1 subjects.

## Aim

This work aimed to evaluate if adolescents with type 1 DM have the same ability to exercise than healthy adolescents.

## Methods

This study was approved by the ethical committee of the Hospital Israelita Albert Einstein **(**Ethical Committee Number: 53638416.9.0000.0071) and free and informed consent was obtained from all participants and their legal representatives.

## Patients

This study enrolled 21 patients with type 1 DM and 16 control subjects, with ages between 10 and 14 years-old. All participants were in stages 2–4 of Tanner’s scale and were considered active or very active according to the IPAQ (International Physical activity questionnaire). To be considered very active, a patient needs to perform vigorous activity at least 3 days/week and for a period longer than 20 min per session. The patients that were considered active performed any combination of walking, moderate intensity or vigorous intensity activities for 5 or more days/week and during more than 150 min/week [14]. The patients with type 1 DM were recruited in the endocrinology services of two hospitals in the city of São Paulo, São Paulo state, Brazil. Each patient invited a friend or a relative of the same age to compose the control group. Patients and control subjects were paired by age, sex, body mass index, weight, height, Tanner’s stage and physical activity level.

The specific inclusion and exclusion criteria for each group were the following:

A. Type 1 DM group: patients had type 1DM for more than 3 years, used a dose of insulin over 0.4 U/Kg/day (a signal of installed type 1 DM) and presented no macrovascular or microvascular chronical complications. Furthermore, the patients had no history of severe and frequent hypoglycemia in the last 6 months (< 60 mg/dl, more than twice a week).
B. Control group: patients did not have any kind of chronic disease.

Controlled and uncontrolled DM:

Within the type 1 DM group, participants were divided in patients with controlled disease (HbA_1c_ < 7.5%) and patients with uncontrolled disease (HbA_1c_ > 7.5%). HbA1c was measured by High Performance Liquid Chromatography (HPLC) in the equipment G8 (Tosoh), which is an assay traceable to the National Glycohemoglobin Standardization Program [15].

## Methods

### Glycemia and blood lactate measurement

Glycemia and blood lactate measurements during the exercise test were performed through capillary puncture of the finger pulp using a 0.5 mm diameter lancet and reactive strips specific for glucose and lactate. Lactate was dosed using the Accutrend^®^ Plus portable lactate meter (Roche, São Paulo, SP, Brazil) and glycemia was measured with the Accu-Chek Active^®^ portable glucose meter (Roche, São Paulo, SP, Brazil). If the patient had glycemia below 70 mg/dl or heart rate above the submaximal value, the exercise test would be interrupted. The submaximal heart rate value was calculated using the formula: (220 − age) × 0.8. In case of hypoglycemia, the patient should immediately ingest 200 ml of juice containing 15 g of sucrose or glucose.

### Incremental submaximal exercise test

Glycemia and blood lactate were measured before the beginning of the incremental submaximal exercise test. The test was performed according to the Balke protocol, in a cycle ergometer (*Technogyn*—Recline model, Gambettola, FC—Italy), with an initial load of 30 W (due to limitation of the minimal load of the bicycle used) and an increase of 25 W every 2-min stage, including recovery time. Heart rate and oxygen saturation were monitored throughout the test with the Oxmax N-600 pulse oximeter (Medtronic—Covidien, Mansfield, MA—EUA). The Borg Rating of Perceived Exertion (RPE) was used to assess the patients fatigue and exhaustion levels. The maximum load was defined as the highest load at which the patient could complete the 2 min of the stage. The test was finalized when the patient interrupted it due to exhaustion or when the patient was not able to keep the speed at 70 rotations per minute. Glycemia and blood lactate were measured in the final 20 s of each stage for determination of LT. LT was reached when blood lactate started to increase exponentially [10].

The estimated VO_2_max (mL/kg/min) was calculated according to the following formula: 200 + (12 × W)/M, where W corresponds to maximum sustained load (watts) and M is the participant’s total body weight (kg).

### Statistical analysis

The power of analysis was calculated using the program PASS 14 (2015), considering the distribution of the sample within the groups and the exact number of subjects in each one of the three groups, using the Kruskal–Wallis test. The level of significance was considered 5%.

Categorical variables were described by absolute frequencies and percentages, while the numerical variables were described by medians and quartiles. The distribution of numerical variables was analyzed by histograms and box-plots and normality was tested using the Shapiro–Wilk normality test.

The characteristics of the participants as well as the data collected during the study were compared between the two groups using the exact Fisher’s test for categorical variables, and the Mann–Whitney or Kruskal–Wallis tests for quantitative variables.

The parameters measured throughout the test were compared among groups by using an adjustment of the Generalized Mixed Models, through a 1st order auto regression model (AR-1) and considering dependence between the performed measurements in the same subject.

The p-values of the tests are presented as results. The SPSS software (IBM, New York, EUA) was employed for all statistical analyses, adopting 5% as significance level.

## Results

Considering the sample size of the groups, we had a statistical power of 80% to detect mean differences of at least one standard deviation (1SD) among most variables compared.

Table 1 demonstrates the characteristics of the participants. HbA_1c_ measurement distinguished two type 1 DM groups (p < 0.001): “controlled type 1 DM” included six adolescents with 6.9% HbA_1c_ (6.5–7.1%) and “uncontrolled type 1 DM” comprised 15 adolescents with 8.9% HbA_1c_ (8.1–9.5%).

**Table 1:** Characteristics of the samples

	Group			*p*
	Controls (n = 16)	Type 1 DM, HbA_1c_ ≤ 7.5% (n = 6)	Type 1 DM, HbA_1c_ > 7.5% (n = 15)	
Age (year)	13.1 (12.8; 13.3)	12.8 (9.7; 13.7)	12.6 (11.4; 13.6)	0.908^#^
Height (cm)	160 (150; 160)	160 (140; 170)	150 (150; 160)	0.773^#^
BMI (kg/m^2^)	19.7 (17.8; 22.8)	20.5 (15.7; 23.0)	19.8 (18.2; 21.7)	0.977^#^
Diabetes duration (months)	–	42.0 (24.0; 84.0)	60.0 (36.0; 96.0)	0.225^$^
HbA_1c_ (%)	–	6.9 (6.5; 7.1)	8.9 (8.1; 9.5)	< 0.001^$^
HbA_1c_ (mmol/mol)	–	52 (48–54)	74 (65–80)	< 0.001^$^
Sex				0.440*
Males	8 (50.0%)	2 (33.3%)	4 (26.7%)	
Females	8 (50.0%)	4 (66.7%)	11 (73.3%)	
Tanner stage				0.214*
2	5 (31.3%)	2 (33.3%)	3 (20.0%)	
3	9 (56.3%)	1 (16.7%)	10 (66.7%)	
4	2 (12.5%)	3 (50.0%)	2 (13.3%)	
Physical activity level				0.807*
Active	11 (68.8%)	3 (50.0%)	10 (66.7%)	
Very active	5 (31.3%)	3 (50.0%)	5 (33.3%)	

Data described by median (1st quartile, 3rd quartile) or n (%)

* Fisher’s exact test

^#^Kruskal–Wallis non-parametric test

^$^Mann–Whitney test

### Responses to exercise

**Fig. 1:**
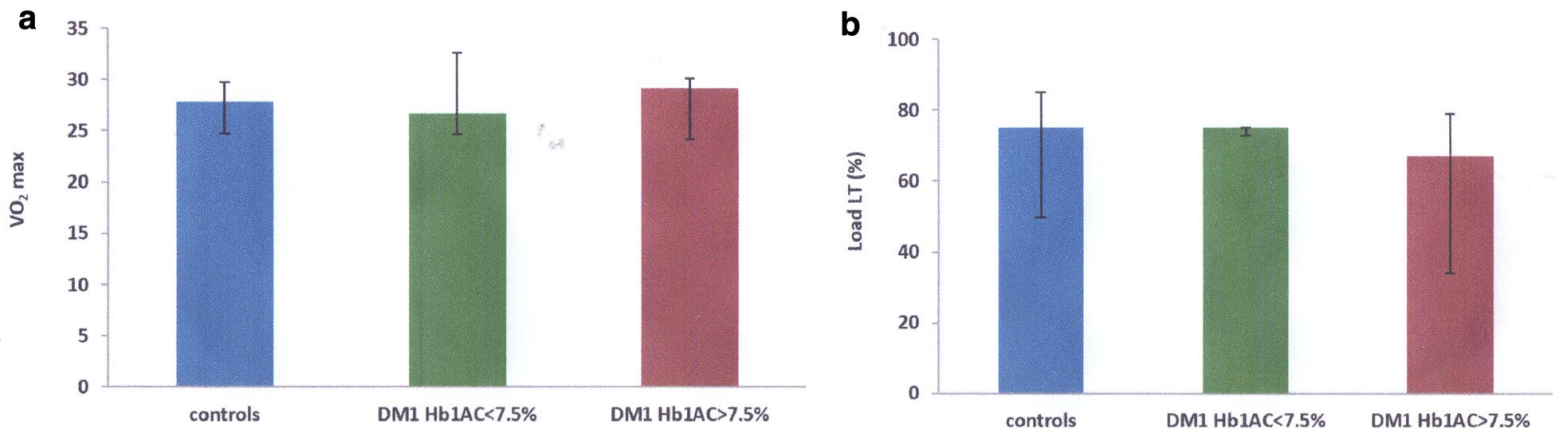
Physical fitness evaluation parameters median VO_2_max **a** and relative load at the lactate threshold (LT) **b** in the three analyzed groups

**Fig. 2:**
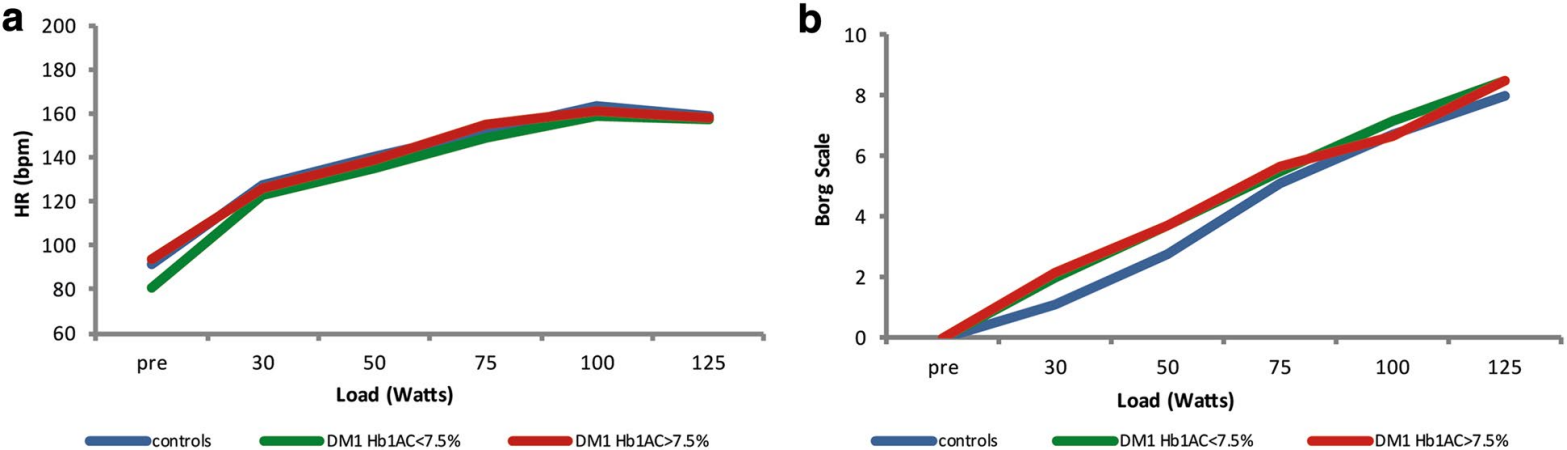
Changes in heart rate (HR) **a** and Borg Scale score **b** during the submaximal exercise test in the three analyzed groups

Borg RPE, VO_2_max, heart rate, maximum load, absolute load to reach LT and relative load to reach LT were all similar among the groups (Table 2 and Fig. 1). Furthermore, all three groups showed similar heart rate behaviour along the test (*p* = 0.49, Fig. 2a), as well as similar Borg RPE (*p* = 0.32, Fig. 2b), indicating similar tolerance to exercise in all three groups.

**Table 2:** Variables for evaluation of physical fitness among the three groups

	Group			p#
	Controls (n = 16)	Type 1 DM, HbA_1c_ ≤ 7.5% (n = 6)	Type 1 DM, HbA_1c_ > 7.5% (n = 15)	
Borg scale max	7.5 (7.0; 9.0)	8.5 (7.0; 9.0)	7.0 (6.0; 9.0)	0.829
VO_2_max	27.8 (24.8; 29.6)	26.6 (25.1; 33.8)	29.1 (24.4; 30.0)	0.977
HR max	167.5 (152.0; 178.0)	162.5 (156.0; 175.0)	165.0 (151.0; 175.0)	0.998
Load max (W)	95.0 (75.0; 100.0)	100.0 (81.2; 100.0)	100.0 (75.0; 100.0)	0.977
Total load of LT (W)	75.0 (50.0; 75.0)	75.0 (75.0; 75.0)	75.0 (50.0; 75.0)	0.377
% load of LT (%)	75.0 (65.0; 100.0)	75.0 (75.0; 77.5)	67.0 (55.0; 77.5)	0.282

Data reported by median (1st quartile, 3rd quartile)

Kruskal–Wallis non-parametric test

All adolescents completed the test and none presented hypoglycemia (< 60 mg/dL) or hyperglycemia (250 mg/dL) (Table 3).

**Table 3:** Mean glycemic values (IC 95%)

Load (W)	Group		
	Controls	Type 1 DM, HbA_1c_ ≤ 7.5%	Type 1 DM, HbA_1c_ > 7.5%
0	95.4 (93.0; 97.8)	141.5 (111.7; 179.3)	184.1 (150.2; 225.6)
30	97.9 (93.7; 102.3)	155.3 (131.1; 184.0)	185.3 (152.7; 224.9)
50	99.3 (94.8; 104.0)	149.8 (118.2; 189.9)	179.2 (147.5; 217.8)
75	90.4 (87.6; 93.3)	151.3 (125.1; 183.0)	174.6 (145.5; 209.5)
100	97.5 (93.1; 102.2)	167.0 (154.1; 181.1)	195.2 (162.5; 234.5)
125	104.5 (94.1; 116.0)	136.6 (127.4; 146.4)	223.5 (185.7; 242.0)

Data reported by mean and 95% confidence intervals

For the 21 patients with type 1 DM, correlation analyses between HbA_1c_, VO_2_max, duration of disease and pre-test glycemic values were performed using the Spearman’s correlation coefficient. A low correlation (0.458) between HbA_1c_ levels and the duration of disease was the only significant correlation found (p = 0.037) (Table 4).

**Table 4:** Correlation coeficients between VO_2_max duration of disease and glycemia

	VO_2_max (mL/kg/min)	Diabetes duration (months)	Pre-test glycemia (mg/dL)
HbA_1c_ (%)	0.094 (p = 0.684)	0.458 (p = 0.037)	0.293 (p = 0.198)
VO_2_max (mL/kg/min)	–	0.236 (p = 0.303)	0.074 (p = 0.750)

Spearman’s correlation coefficient

## Discussion

In this study, we compared the physical fitness of adolescents with controlled and uncontrolled type 1 DM with that of healthy age-matched control subjects, using the incremental submaximal exercise test. The present study evaluated the anaerobic threshold by measuring lactate blood levels, and estimated VO_2_max considering the maximum load.

Studies on the physical fitness of adolescents with type 1 DM compared to healthy control subjects have yielded conflicting results [16–19]. Whereas Komtsu et al. reported that patients with DM have lower VO_2_max and an earlier exhaustion time than healthy controls [16], Heyman and Adolfsson found no significant differences in the aerobic exercise capacity of these two groups in their analysis of VO_2_max [18, 19]. The results of present study are in accordance with Heyman and Adolfsson’s, as we found no differences between patients with type 1 DM, whether controlled or uncontrolled, and healthy subjects in either VO_2_max, maximum load during the test, or absolute and relative load at the lactate threshold. Individual physical fitness is dependent on a complex hormonal regulation that requires insulin, glucagon, adrenaline and noradrenaline. This hormonal control is directly influenced by glucose usage and by the catecholamine levels that vary according to the exercise intensity. Subjects with type 1 DM have a higher tendency to present hypoglycemia, due to their compromised negative feedback response [11]. However, if the administrated amount of insulin is correct, no hypoglycemia should occur during exercise, resulting in a normal physical fitness [19].

Besides hypoglycemia, hyperglycemia might also occur in patients with type 1 DM performing high intensity exercises. The release of adrenaline in this type of exercise increases heart rate and stimulates hepatic glycogenolysis. This leads to an increase in blood glucose levels that may cause early exhaustion [20]. However, we detected neither hyper- nor hypoglycemia episodes during the incremental submaximal exercise test, suggesting that the patients with type 1 DM were able to regulate their blood glucose levels even under such conditions. This implies that the amount of insulin administrated to the patients during the test was adequate, and did not compromise their physical fitness.

Williams et al. observed a higher heart rate increase in response to exercise in patients with type 1 DM than in healthy subjects, and attributed this to a lower physical fitness in patients with type 1 DM than in healthy individuals [21]. In contrast, in our study both groups presented similar heart rate and Borg RPE at all stages of the test, indicating similar aerobic physical capacities in control subjects and in adolescents with controlled or uncontrolled type 1 DM. An important aspect of our study was the level of physical activity and the cardiovascular performance of the participants. The adolescents included in the study had a homogeneous profile. All participants had ages between 10 and 14 years-old and were in Tanner’s stages 2–4. They all performed physical activities for at least 3 h/week and were defined as active or very active by the IPAQ [14]. Moreover, only patients without diabetes-associated complications and additional chronic diseases were included. Maggio et al. have described that adolescents with chronic diseases (type 1 DM, obesity and idiopathic juvenile arthritis) perform less physical activities when compared to controls, which impacts their physical fitness [22]. Our patients regularly practiced physical activities, which improves their cardiovascular function and increases tolerance to effort, leading to higher VO_2_max levels and a delayed lactate threshold.

In this study, the values of HbA_1c_ were not associated with VO_2_max, which had similar values in uncontrolled patients with type 1 DM, controlled patients with type 1 DM and control subjects. This suggests that HbA_1c_ does not affect the aerobic physical fitness of the patients. However, previous studies have described a negative association between HbA_1c_ values and VO_2_max. In all of them, the VO_2_max was lower in DM patients than in control subjects [13, 23, 24]. Cuenca-Garcia et al. evaluated the physical activity of patients with type 1 DM and control subjects using accelerometry and tested their cardiovascular function using the incremental submaximal exercise test. These parameters were then related to HbA_1c_ levels in patients with type 1 DM. As in our study, no significant correlation was identified [25]. Nonetheless, it is important to notice that the study by Cuenca-Garcia identified a relation between moderate to vigorous exercise and improved glycemic control. In another study, Roberts et al. also found no association between HbA_1c_ levels and VO_2_max, but, contrarily to the work of Cuenca-Garcia, no association between glycemic control and intense, vigorous exercise was described. Altogether, these data suggest that physical activity is more important for a good cardiopulmonary performance of patients with type 1 DM than HbA_1c_ levels [26].

Based on a significant difference in HbA_1c_ values (p < 0.001), the adolescents enrolled in the study were divided in two groups: those with controlled disease (HbA1c < 7.5%; median 6.9%, 52 mmol/mol) and those with uncontrolled disease (HbA1c > 7.5%; median 8.9%, 74 mmol/mol). The HbA_1c_ median difference between both groups is in accordance with the definition used by Nguyen et al., that defined controlled patients with type 1 DM has having HbA_1c_ below 7.5%, while uncontrolled patients had values over 9.0% [27]. One of the limitations of this study was not to have performed patient recruitment based on HbA_1c_ levels.

Determination of the anaerobic threshold using blood lactate levels is a practical, reliable and easy-to-use technique that dispenses with the infrastructure needed for ergospirometry. This was the first study using blood lactate dosage to evaluate the anaerobic threshold in adolescents with type 1 DM. Studies performed over a decade ago have validated this technique, by showing a tight relation between LT and thresholds measured by ergospirometry [28]. Ahmaidi et al. have identified a strong correlation between thresholds and blood lactate levels in young athletes (r = 0.90, p < 0.001) [29]. More recently, Ribeiro et al., assessing the anaerobic threshold through ventilatory and metabolic parameters in 28 professional swimmers, reported that cardiac frequency and swimming speed were similar for both evaluation methods, indicating that ventilatory threshold and lactate threshold yield similar results [30].

Regular exercise has important benefits for everyone. The possibility of hypoglycemia occurring during or after exercise tends to make averts patients with type 1 DM avoid performing physical activities. Undergoing an incremental submaximal exercise test is therefore of major importance, since it allows for the definition of individual exercise intensity that ensures the safety of the prescribed physical activity by reducing the risk of hypoglycemia events. The confirmation that adolescents with type 1 DM have the same aerobic exercise capacity as healthy subjects allows for an increase in the opportunities of patients with type 1 DM to engage in group physical activities, favoring social life and improving the quality of life of the patients.
